# Predicting p53 Status in IDH‐Mutant Gliomas Using MRI‐Based Radiomic Model

**DOI:** 10.1002/cam4.71063

**Published:** 2025-08-01

**Authors:** Jiamin Li, Zhihong Lan, Xiao Zhang, Xiaoyun Liang, Hanwei Chen, Xiangrong Yu

**Affiliations:** ^1^ Department of Radiology Zhuhai People's Hospital, Zhuhai Hospital Affiliated With Jinan University Zhuhai China; ^2^ Department of Radiology Guangzhou Panyu Health Management Center (Guangzhou Panyu Rehabilitation Hospital) Guangzhou China; ^3^ Medical AI Lab, The First Hospital of Hebei Medical University Shijiazhuang China; ^4^ Hebei Provincial Engineering Research Center for AI‐Based Cancer Treatment Decision‐Making, The First Hospital of Hebei Medical University Shijiazhuang China; ^5^ Department of Oncology The First Hospital of Hebei Medical University Shijiazhuang China; ^6^ Institute of Research and Clinical Innovations, Neusoft Medical Systems Co. Ltd Guangzhou China

**Keywords:** contrast‐enhanced T1WI, glioma, IDH‐mutant, p53 status, radiomics

## Abstract

**Objectives:**

Accurate and noninvasive detection of p53 status in isocitrate dehydrogenase mutant (IDH‐mt) glioma is clinically meaningful for molecular stratification of glioma, yet it remains challenging. We aimed to investigate the diagnostic efficacy of radiomics utilizing pre‐surgery contrast‐enhanced T1‐weighted imaging (CE‐T1WI) for predicting p53 status in IDH‐mt gliomas.

**Methods:**

A total of seventy‐eight patients with pathologically confirmed IDH‐mutant glioma were admitted to our institution between January 2011 and October 2018. For each patient, three types of volumes of interest (VOIs) were segmented: (i) VOI^T^: the entire tumor (including the necrotic area within the tumor); (ii) VOI^PE^: the peritumoral edema; (iii) VOI^T + PE^: the entire tumor and peritumoral edema. A total of 962 radiomic features were extracted for each VOI, followed by feature selection and modeling (Rad_VOI^T^, Rad_VOI^PE^, and Rad_VOI^T + PE^ models) using machine learning algorithms. A nomogram was developed to integrate significant clinical factors and radiomic predictors for p53 status prediction. Akaike Information Criterion (AIC) was leveraged as the stopping rule. The predictive performance of the models was evaluated using receiver operating characteristic (ROC) curve analysis.

**Results:**

The VOI^PE^ model, which included the eight best‐performing features, demonstrated the highest predictive performance among the three VOI‐based models, with AUCs of 0.811 (95% CI: 0.782–0.840) and 0.810 (95% CI: 0.786–0.834) in the training and validation cohorts, respectively. Age was identified as the only significant clinical factor and was combined with the Rad‐scores from VOI^T^, VOI^PE^, and VOI^T + PE^ to construct a clinical‐radiomics nomogram with the most notable discriminative ability for p53 status (AIC = −120.19). The AUCs for this nomogram were 0.969 (95% CI: 0.942–0.996) in the training cohort and 0.929 (95% CI: 0.898–0.960) in the validation cohort.

**Conclusion:**

The CE‐T1WI‐based radiomic model can noninvasively predict p53 mutation status in IDH‐mt gliomas. The textural differences of peritumoral edema may more accurately reflect the underlying tumor heterogeneity associated with p53 status.

## 1 Introduction

Glioma is the most common primary intracranial tumor, accounting for approximately 81% of malignant brain tumors [1]. Currently, the standard treatment for glioma includes maximum safe resection followed by radiotherapy and chemotherapy [2]. The revised 2016 edition of the World Health Organization (WHO) Classification of Tumors of the Central Nervous System (CNS) incorporates molecular parameters alongside histology to define the primary tumor categories for the first time [1, 3, 4]. Current postoperative treatment strategies for major glioma entities are based on this classification, underlining the significant role of molecular biomarkers in the selection of treatment protocols [5].

In 2021, the WHO updated the classification of CNS tumors, mandating assessment of both molecular and neuropathological profiles [6]. Isocitrate dehydrogenase (IDH) and 1p/19q status serve as crucial molecular markers in diffuse astrocytomas, some glioblastomas, and medulloblastomas [4, 7, 8]. IDH gene mutations affect tumor metabolism, microstructural changes, angiogenesis, and significantly influence patient survival and treatment response. Houillier et al. [9] suggested that patients with IDH‐mutant (IDH‐mt) gliomas experience enhanced chemotherapy responses. The p53 gene functions as a tumor suppressor and transcription factor; mutations not only abolish wild‐type functions but also gain oncogenic functions, promoting cell proliferation, invasion, and migration [10, 11]. Fu et al. [12] found higher mutant p53 prevalence in high‐grade gliomas compared to low‐grade ones, implying its association with glioma malignancy. Previous studies have suggested elevated Epidermal Growth Factor Receptor (EGFR) expression in IDH and p53 mutated gliomas, correlating with increased vascularity, radiation resistance, and distinct imaging features [13, 14]. Notably, the p53 status in IDH‐mutant gliomas plays a crucial role in tumor progression and response to temozolomide treatment [3, 15], requiring histological specimens obtained via surgical resection or stereotactic biopsy for accurate detection [3]. Noninvasive methods to identify p53 mutation status become critical when surgery is not viable due to poor physical performance or the tumor being located in vital brain regions.

In recent years, the rapid advancement of artificial intelligence (AI) has revolutionized various fields, including medicine. Machine learning (ML) has emerged as a powerful tool for developing AI‐based predictive models that assist clinicians in making more informed decisions [16, 17, 18]. Radiomics, which involves the application of automated data characterization algorithms to convert image data into high‐resolution spatial feature data for downstream analysis, has benefited significantly from the integration of ML techniques [19]. By leveraging machine‐learning algorithms, researchers can identify AI‐driven radiomic signatures that capture the intrinsic heterogeneity of tumors and provide valuable prognostic information. This technique is particularly effective in capturing the heterogeneity of IDH‐mutated gliomas and correlating these variations with underlying gene expression patterns. For example, Liu et al. [20] explored the specific radiomic characteristics of IDH mutation in low‐grade gliomas (LGGs) using T2‐weighted imaging (T2WI). The study revealed that the gene alterations and biological behaviors of IDH‐mt and IDH wild‐type (wt) LGGs, including immune response, vascular development, adhesion, and proliferation, could be manifested through radiological imaging. Martin et al. [21] found that contrast‐enhanced T1‐weighted imaging (CE‐T1WI) performed optimally for IDH typing of glioblastoma using radiomic analysis based on filter histogram, achieving 92% accuracy, 100% sensitivity, and an area under the curve (AUC) of 0.945. As research progresses, deep learning methods have shown superiority over traditional machine learning methods in predicting the genetic and molecular biology of tumors based on MRI. Convolutional neural networks (CNNs) can automatically extract features from raw image data, often outperforming traditional ML techniques in terms of accuracy and robustness. A new fully automated deep learning method based on T2WI has been employed to classify IDH mutation status in glioma, achieving higher predictive performance. Specifically, the average cross‐validation accuracy, sensitivity, specificity, and AUC of T2‐Network in predicting IDH mutation status were 87.6%, 0.76, 0.91, and 0.89 respectively [22]. While previous studies have demonstrated the significant role of radiomics in predicting glioma genes and revealing biological behavior, few have focused on the p53 status in IDH‐mutated gliomas.

The objective of this study is to investigate the relationship between radiomic signatures and the p53 phenotype within the framework of IDH mutations, thereby advancing the application of radiomics in clinical decision‐making for glioma molecular classification. Additionally, the study compares the predictive efficacy of radiomic models based on volumes of interest (VOIs) of the entire tumor, peritumoral edema, and both regions in determining p53 mutation status.

## 2 Materials and Methods

### 2.1 Patients

Institutional review board approval was obtained for this retrospective study, with a waiver of written informed consent from the patients. A total of 78 patients (mean age, 40.77 years ±8.71; range, 23–60 years; male/female, 51/27) with histologically confirmed IDH‐mutant glioma between January 2011 and October 2018 were enrolled. The patients were divided into a training cohort (*n* = 51) for the development of a CE‐T1WI‐based radiomic signature and a validation cohort (*n* = 27) for performance evaluation. Details of patient inclusion and exclusion criteria are summarized in Supporting Information.

### 2.2 Genetic Phenotypes

In our study, the determination of IDH and p53 status was performed through gene sequencing. Any identified nucleotide changes that led to amino acid substitutions or frameshift mutations were classified as mutations. Consequently, samples were categorized based on the presence or absence of mutations: those with detectable mutations were classified as mutant, while those without mutations were classified as wild‐type. Using this approach, we classified glioma samples into two groups based on p53 status: mutant and wild‐type. Similarly, IDH status was categorized into mutant and wild‐type groups, following the same criteria. It is important to note that our study specifically focused on IDH‐mutant gliomas; therefore, all patients included in our analysis were confirmed to have IDH mutations.

### 2.3 MRI Acquisition and Processing

All patients underwent CE‐T1WI scans using a 3.0 T MRI scanner (Siemens Medical Solutions). The acquisition parameters were as follows: Repetition Time/Echo Time (TR/TE) = 1900/2.93 ms; Field of View (FOV) = 218 × 250 mm^2^; matrix = 256 × 215; slice thickness = 1 mm; slice gap = 0.2 mm. CE‐T1WI images that met the inclusion criteria were retrieved from the picture archiving and communication system.

The MRI standardization process included offset correction, gray level normalization, and voxel size resampling. First, MRI offset correction was performed using the N4 algorithm embedded in 3D Slicer version 4.11.0 (https://www.slicer.org). Subsequently, gray level normalization was applied to map the gray values of the CE‐T1WI scans to a range of 0–255. Finally, the image voxel size was uniformly resampled to 1 × 1 × 1 mm^3^ using the cubic B‐spline interpolation algorithm, which minimized feature differences caused by varying reconstruction slice thicknesses and pixel sizes.

### 2.4 Tumor Segmentation and Reproducibility Evaluation

The VOI segmentation was performed using ITK‐SNAP version 3.6.0 (https://www.itk‐snap.org). Three types of VOIs were defined as follows: (i) VOI^T^, volume of entire tumor (including necrotic areas); (ii) VOI^PE^, volume of peritumoral edema; and (iii) VOI^T + PE^, volume of entire tumor plus peritumoral edema. Two experienced radiologists specializing in brain tumor imaging (with 10 and 6 years of experience, respectively) independently conducted the double‐blind VOI segmentation. To address variations in tumor area definitions among radiologists, discrepancies in segmentation were resolved by consulting a senior radiologist (with 20 years of experience). Additionally, one of the radiologists re‐segmented the VOIs after four‐week washout period to assess individual subjective influences. Subsequently, using the radiomic features extracted, we evaluated inter‐ and intra‐observer segmentation reproducibility using the intra‐class correlation coefficient (ICC). A cutoff value of 0.75 was applied to select a stable and robust set of features for further feature selection and modeling processes.

### 2.5 Radiomic Feature Extraction

PyRadiomics [23] was utilized for radiomic feature extraction. According to the customized extraction settings detailed in Supporting Informations, we extracted a total of 962 radiomic features from each VOI using filters applied to the original and derived CE‐T1WI images. Two primary types of filters were: the Laplacian of Gaussian (LoG) filter, which highlights areas of gray level change and categorizes textures as fine or coarse based on the sigma size, and wavelet filtering, which provides multiscale information by applying either a high‐pass or low‐pass filter in each of the three dimensions. The extracted radiomic features include the following categories: (i) shape‐based features (*n* = 14); (ii) histogram‐based first‐order features (*n* = 72); (iii) matrix‐based texture features: gray‐level co‐occurrence matrix (GLCM) features (*n* = 96), gray‐level run length matrix (GLRLM) features (*n* = 64), gray‐level size zone matrix (GLSZM) features (*n* = 64), and neighborhood gray‐tone difference matrix (NGTDM) features (*n* = 20); and (iv) wavelet‐filtered features (*n* = 632). Details of the feature types can be found in Supporting Informations.

### 2.6 Radiomic Feature Selection and Signature Construction

The Synthetic Minority Oversampling Technique (SMOTE) strategy [24] was initially employed to balance positive and negative samples in the training cohort. For the set of stable and robust features (inter‐ICCs ≥ 0.75 and intra‐ICCs ≥ 0.75) obtained, we performed Z‐score normalization to standardize the feature values to a normal distribution with zero mean and unit standard deviation, thereby mitigating the impact of varying feature scales. Subsequently, Pearson correlation coefficient (PCC) analysis was conducted to ensure strong correlations with labels while maintaining feature independence. For paired features with PCCs ≥ 0.95, the feature exhibiting the stronger correlation with p53 status (higher correlation coefficient) was retained, while the other was excluded. The feature set, reduced in redundancy through this process, underwent additional filtering using the Kruskal–Wallis test, Relief [25], or recursive feature elimination (RFE) algorithm. In this study, cross‐validation using the leave‐one‐out method was employed to select optimal hyperparameters and determine the final radiomic signature from the selected representative features. Accordingly, five classifiers were compared to develop the optimal radiomic signature: random forest (RF), support vector machines (SVM), logistic regression (LR), least absolute shrinkage and selection operator (LASSO), and Gaussian process (GP). Once the final radiomic signature was identified, we computed the radiomic score (rad‐score) for each patient, serving as a quantitative tool to assess individual differences and as an independent factor for fusion analysis. The performance of the radiomic signature was evaluated using an in‐house validation cohort, where rad‐scores were concurrently computed for patients in this group. For clarity, we labeled the rad‐scores derived from the various radiomic signatures as Rad_VOI^T^, Rad_VOI^PE^, and Rad_VOI^T + PE^, respectively.

### 2.7 Clinical‐Radiomics Nomogram Building and Clinical Use Assessment

To rule out the potential influence of clinical risk factors, a stepwise multivariate logistic regression analysis with a backward search method was performed for three clinical risk factors (age, sex, and pathological stage) and the three rad‐scores. The likelihood ratio test and Akaike information criterion (AIC) were employed as the stopping rule. The model with the lowest AIC score is considered the best among the candidate models, as it provides the optimal trade‐off between fit and complexity. A clinical‐radiomics nomogram was developed by incorporating the significant factors, thus providing clinicians with an efficient tool for predicting p53 status. Calibration curves were plotted to assess the agreement between the estimated probability and the actual outcomes, with the Hosmer–Lemeshow test serving as the benchmark. The process was subjected to bootstrapping validation (1000 bootstrap resamples) to calculate a relatively corrected performance. The degree of overlap between the calibration curve and the diagonal in the graph reflects the predictive accuracy of the nomogram. In addition, decision curve analysis (DCA) was conducted to evaluate the clinical utility by calculating the net benefits for a range of threshold probabilities.

### 2.8 Statistical Analysis

A *t*‐test or Mann–Whitney *U* test was appropriately employed to assess statistical variances in continuous variables, while the Chi‐Square test was utilized to examine differences in categorical variables between the p53‐wt and p53‐mt groups. The performance of the radiomic signatures was quantified by the area under the AUC. Moreover, sensitivity, specificity, positive predictive value (PPV), and negative predictive value (NPV) were calculated at a cut‐off value that maximized the Youden index. The radiomic analysis was performed using Python version 3.7.6. A two‐tailed *p* value < 0.05 was considered statistically significant.

## 3 Results

### 3.1 Patient Characteristics

The clinical characteristics of patients in the training and validation cohorts are displayed in Table 1. The majority of enrolled patients were female (45 out of 78). Although the age range spanned over 30 years in both cohorts, the majority of patients were between 40 and 50 years old. However, no statistically significant differences were observed between the p53‐wt and p53‐mt groups in terms of all characteristics (*p* > 0.05).

**TABLE 1:** Clinical characteristics of patients.

Cohorts	p53 status	Grades			Age (years)	*p*	Sex (female/male)	*p*
		II	III	IV				
Training cohort	p53‐wt	7	1	5	40.31 ± 11.56	0.375	11/2	0.082
	p53‐mt	14	6	18	40.50 ± 7.20		16/22	
Validation cohort	p53‐wt	5	1	1	44.43 ± 6.68	0.293	5/1	0.756
	p53‐mt	5	3	12	40.30 ± 9.31		13/7	

### 3.2 Tumor Segmentation Reproducibility Evaluation

The inter‐observer ICCs for evaluating individual subjective influence showed varying levels of reliability across different thresholds (ICCs ≥ 0.75, *n* = 897; ICCs ≥ 0.80, *n* = 822; ICCs ≥ 0.85, *n* = 740; ICCs ≥ 0.90, *n* = 542). Furthermore, the agreement in tumor area delineation between two radiologists yielded similar results (ICCs ≥ 0.75, *n* = 895; ICCs ≥ 0.80, *n* = 830; ICCs ≥ 0.85, *n* = 746; ICCs ≥ 0.90, *n* = 565). Considering the impact of inter‐ and intra‐observer segmentation reproducibility, we identified a set of relatively stable and robust features with a cutoff value of 0.75 (ICC ≥ 0.75, *n* = 886; ICC ≥ 0.80, *n* = 804; ICC ≥ 0.85, *n* = 716; ICC ≥ 0.90, *n* = 509). Consequently, subsequent feature selection and development of the radiomic signature were based on the selected 886 features.

### 3.3 Radiomic Feature Selection

**FIGURE 1:**
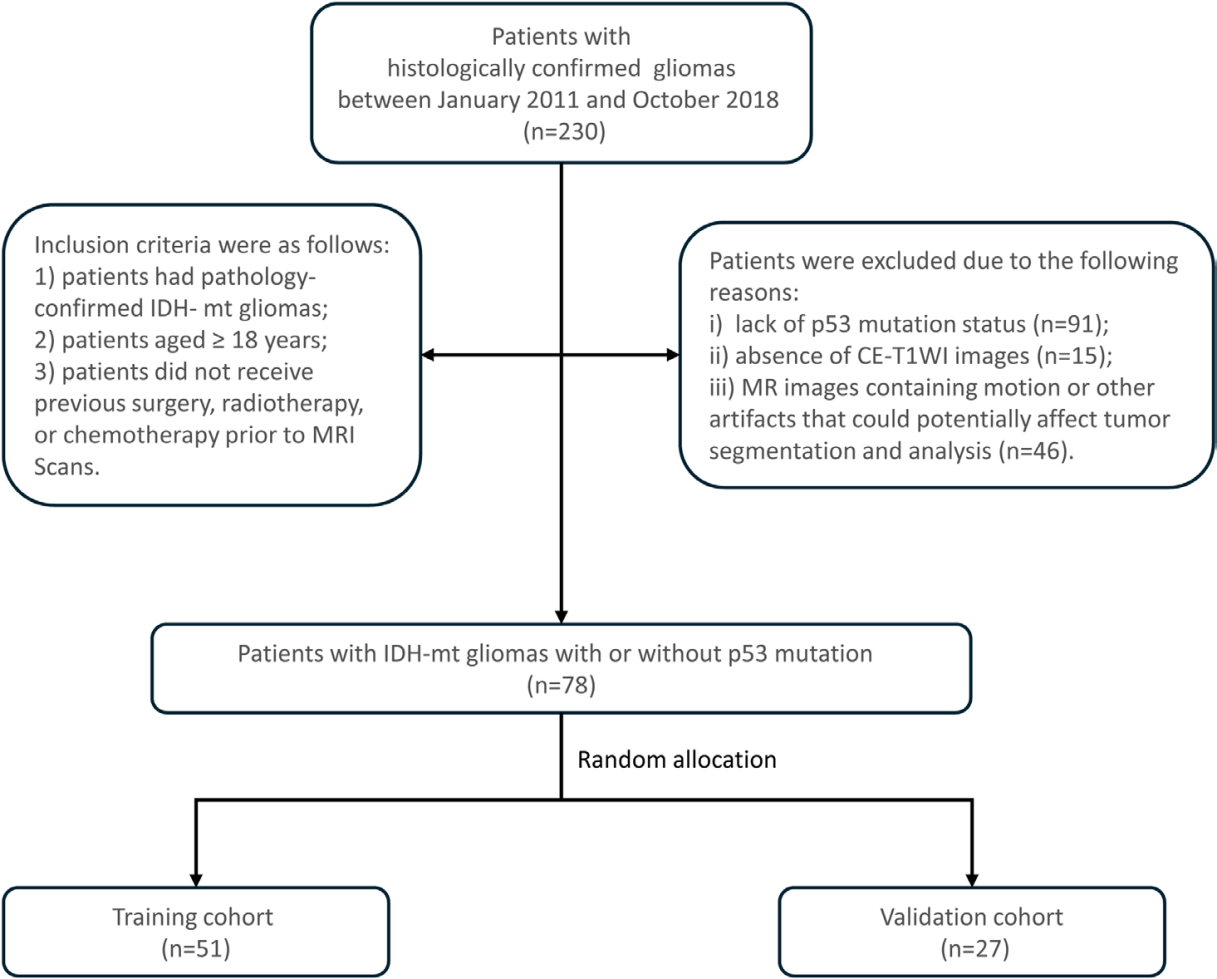
Flowchart of study enrollment.

PCC analysis showed high inter‐dependencies among radiomic features, retaining 601, 592, and 612 features extracted from VOI^T^, VOI^PE^, and VOI^T + PE^, respectively. After that, 2 and 6 features were selected using RFE for building the radiomic signatures from the VOI^T^ and VOI^T + PE^, while 8 features selected via Kruskal–Wallis analysis were used for developing the VOI^PE^ signature. Detailed features are provided in Table 2. To illustrate, feature maps of texture features identified from VOI^T^ and VOI^PE^ for two randomly selected patients are shown in Figure 1. The values of these texture features varied between the p53‐wt and p53‐mt groups, with VOI^PE^‐derived features showing a strong capability to identify p53 status.

**TABLE 2:** Highly discriminative radiomic features in various models.

VOI type	Image type	Feature type	Feature name
VOI^T^	Wavelet‐HHH	First‐order	Median
	Wavelet‐HHH	GLCM	Correlation
VOI^PE^	Original	GLRLM	Run Entropy
	LoG‐2 mm	GLRLM	Run Entropy
	LoG‐4 mm	GLSZM	Gray Level Non‐Uniformity Normalized
	LoG‐6 mm	GLCM	Idmn
	LoG‐6 mm	GLCM	Idn
	LoG‐6 mm	NGTDM	Contrast
	Wavelet‐LHL	First‐order	Median
	Wavelet‐LLL	GLRLM	Long Run High Gray Level Emphasis
VOI^T + PE^	LoG‐4 mm	GLSZM	Gray Level Non‐Uniformity
	LoG‐4 mm	GLSZM	Low Gray Level Zone Emphasis
	LoG‐6 mm	GLSZM	Low Gray Level Zone Emphasis
	Wavelet‐HLH	NGTDM	Strength
	Wavelet‐HHH	First‐order	Median
	Wavelet‐HHH	First‐order	Skewness

### 3.4 Predictive Performance of the Radiomic Signatures

**FIGURE 2:**
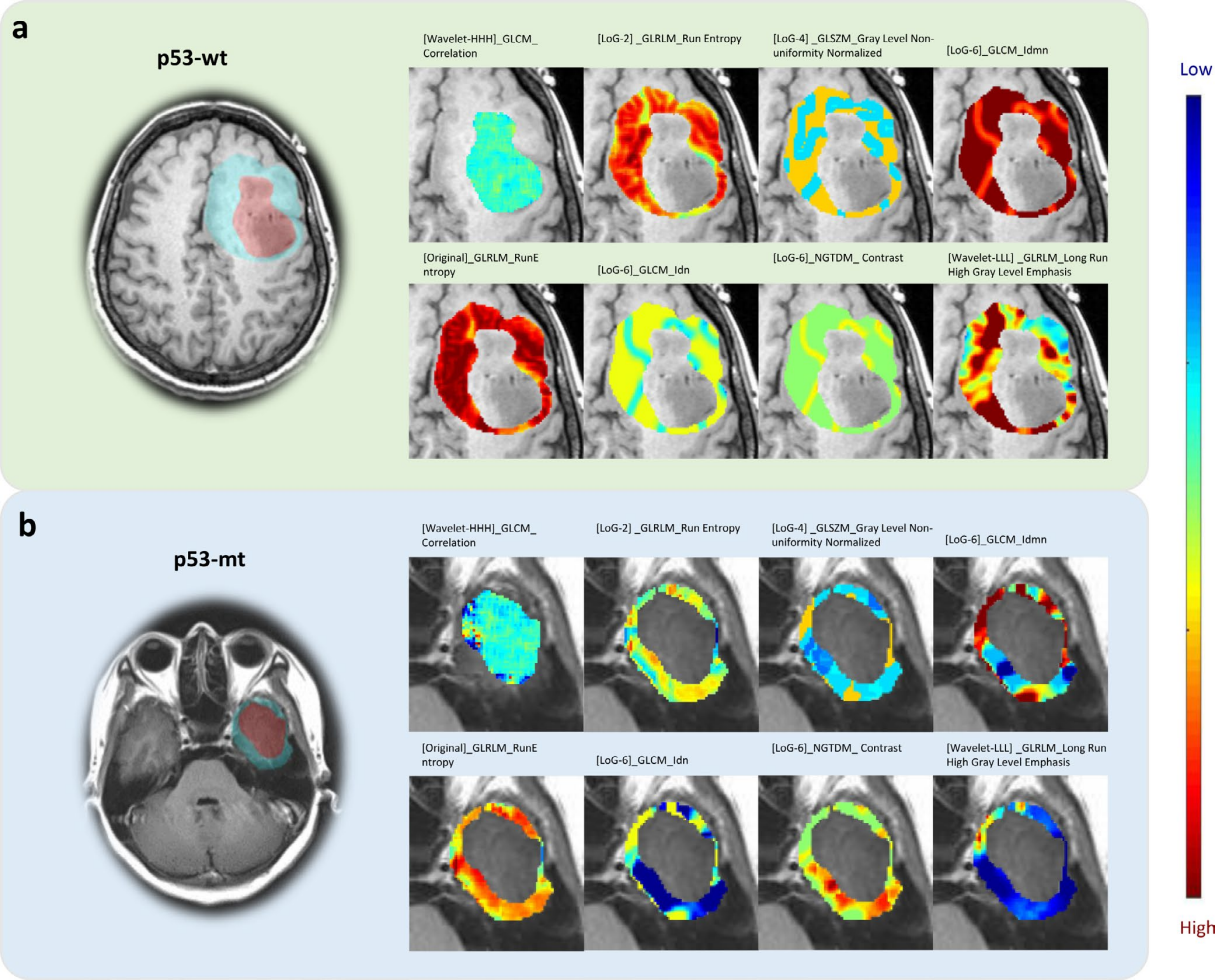
Texture feature maps for the selected radiomic signatures from VOI^T^ and VOI^PE^. (a) a 45‐year‐old woman diagnosed with p53‐wt in grade II glioma; (b) a 40‐year‐old woman diagnosed with p53 mutation in grade II glioma. Note that the MRI slice shown is the slice with the largest segmentation region. The segmented areas filled with red and blue represent the slice of VOI^T^ and VOI^PE^, respectively.

For the VOI^T^ signature, the 2 selected radiomic features were modeled using a GP classifier, yielding an AUC of 0.770 (95% CI: 0.737–0.803) and 0.770 (95% CI: 0.741–0.799) in the training and validation cohorts, respectively. The 8 best‐performing features from VOI^PE^ were modeled using LR, showing a more prominent AUC of 0.811 (95% CI: 0.782–0.840) and 0.810 (95% CI: 0.786–0.834) in the training and validation cohorts, respectively. Using a SVM classifier, the VOI^T + PE^ signature with 6 features also showed satisfactory discriminative ability for p53 prediction in both cohorts (AUC: 0.846, 95% CI: 0.811–0.881 and 0.778, 95% CI: 0.749–0.807, respectively). This indicates the potential value of tumor margin texture in differentiating p53 status. The radiomic scores for each patient can be calculated using the code available on GitHub (https://github.com/hbydyyail/p53_status_prediction.git). Additional evaluation metrics for the signatures are presented in Table 3. Furthermore, the corresponding rad‐scores for the two p53 groups from the three signatures are plotted in Figure 2 to visualize the predictive differences (all *p* < 0.05).

**TABLE 3:** Evaluation metrics of the models.

Cohorts	Models	AUC	*p*	Sensitivity	Specificity	NPV	PPV
Training cohort	VOI^T^	0.770	Ref.	0.676	0.786	0.893	0.478
	VOI^PE^	0.811	0.042*	0.784	0.643	0.853	0.529
	VOI^T + PE^	0.846	0.008*	0.919	0.786	0.919	0.786
	Nomogram	0.969	< 0.001*	0.865	1.000	1.000	0.737
Validation cohort	VOI^T^	0.770	Ref.	0.619	0.667	0.867	0.333
	VOI^PE^	0.810	0.054	0.619	1.000	1.000	0.429
	VOI^T + PE^	0.778	0.774	0.762	0.667	0.889	0.444
	Nomogram	0.929	< 0.001*	0.667	1.000	1.000	0.462

* Indicates that the *p*‐value for the deleong test is < 0.05.

### 3.5 Clinical‐Radiomics Nomogram Building and Evaluation

**FIGURE 3:**
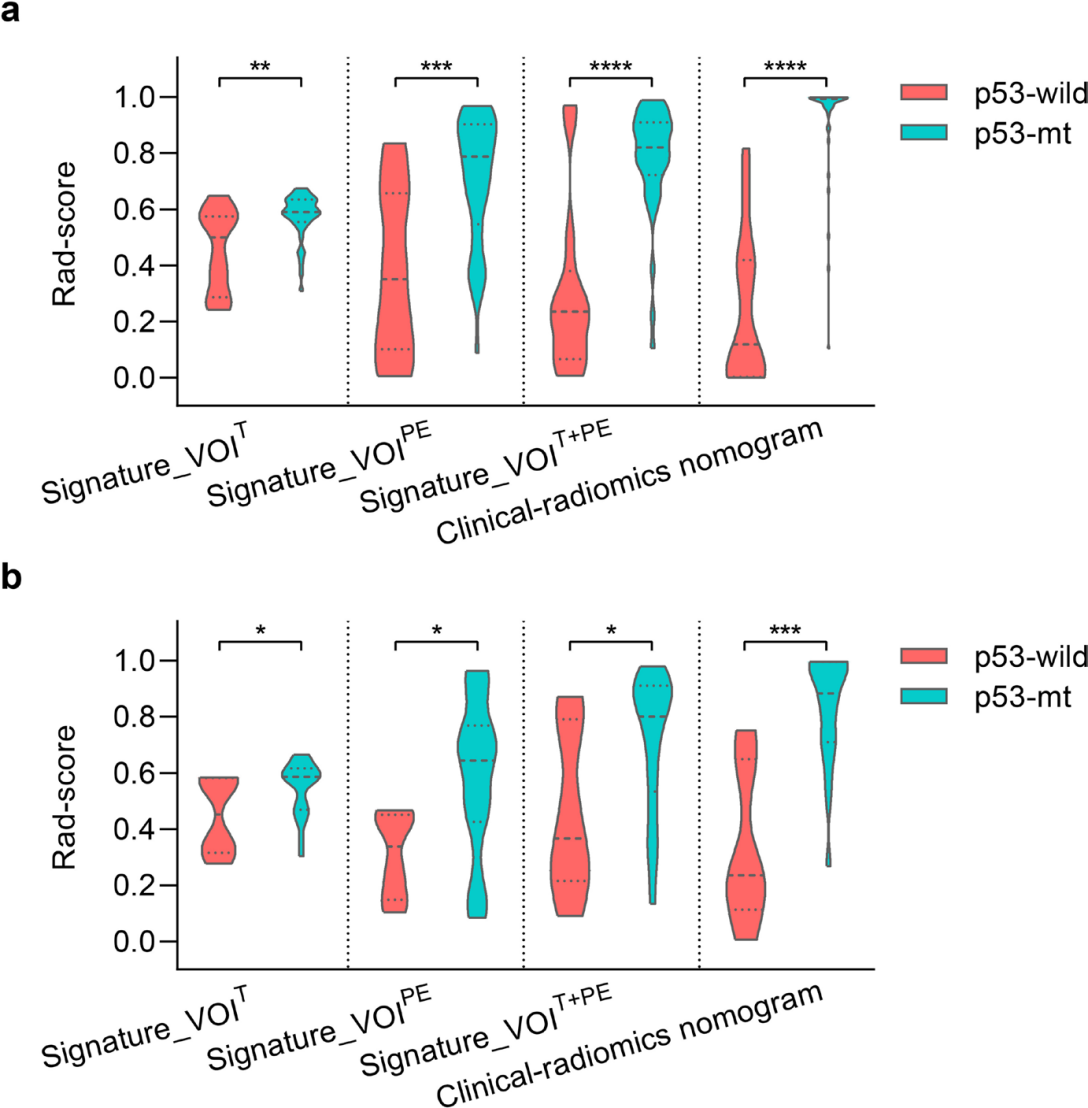
Rad‐scores of Rad_VOI^T^, Rad_VOI^PE^, Rad_VOI^T + PE^, and clinical‐radiomics nomogram between the p53‐wt and p53‐mt groups. (a) training cohort; (b) validation cohort. The asterisk (*) indicates a significant difference.

**FIGURE 4:**
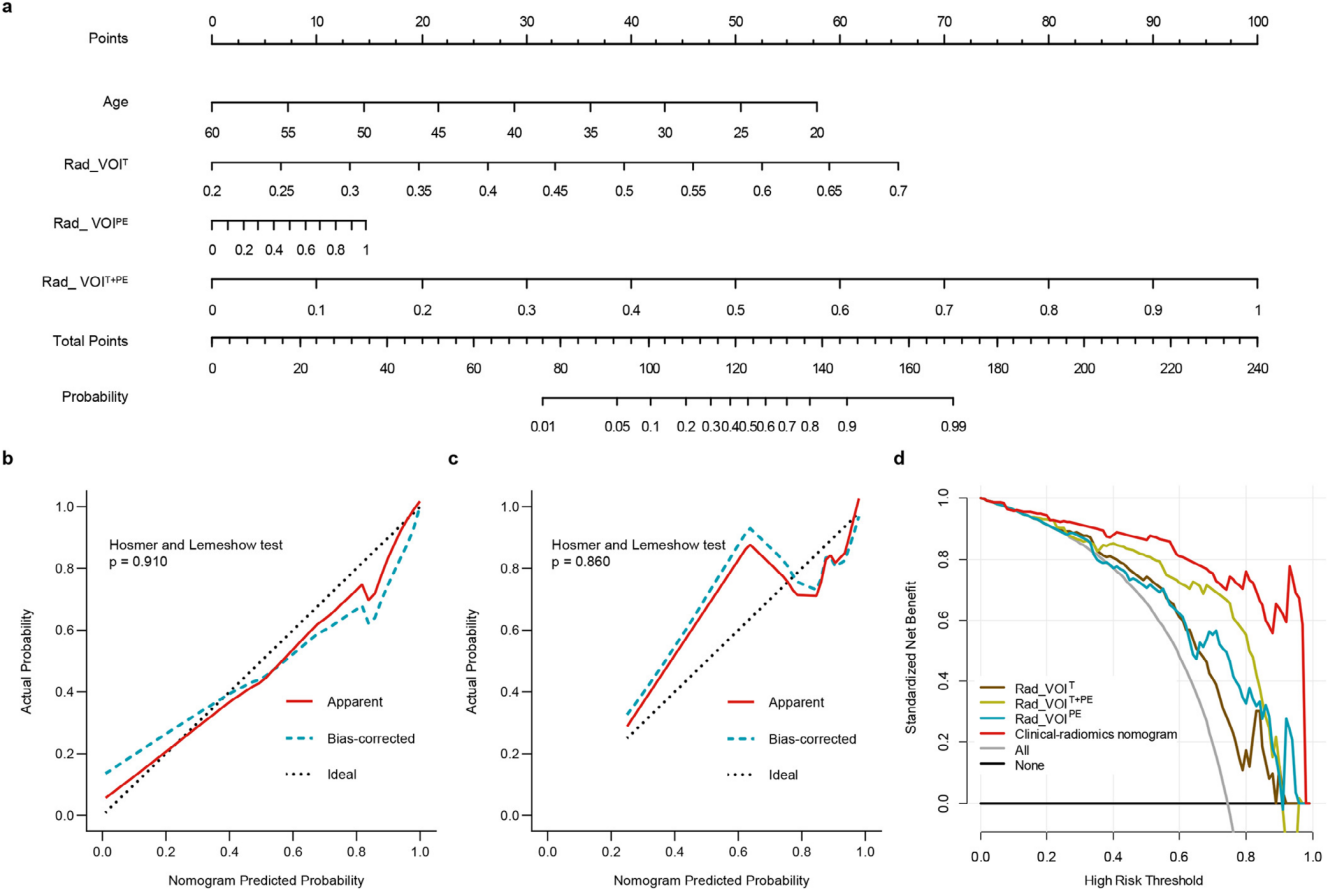
Clinical‐radiomics nomogram. (a) nomogram; (b) calibration curve for the training cohort; (c) calibration curve for the validation cohort; (d) decision curve analysis on the whole cohort.

Stepwise logistic regression analysis identified age as the only clinical predictor (Odds ratio, 0.87; 95% CI: 0.737–1.023). A clinical‐radiomics nomogram was then developed by combining age and the rad‐scores from the three radiomic signatures of VOI^T^, VOI^PE^, and VOI^T + PE^ (AIC = −120.19, Figure 3a). The results suggested that the nomogram had superior performance compared to the individual radiomic signatures alone, with AUCs of 0.969 (95% CI: 0.942–0.996, all *p* < 0.05) and 0.929 (95% CI: 0.898–0.960, all *p* < 0.05) in the training and validation cohorts, respectively (Table 3). The prediction probability from the nomogram (nom‐score) was defined as: nom‐score = −6.118 − 0.141*Age + 12.818*Rad_VOI^T^ + 9.763*Rad_VOI^T + PE^ + 1.438*Rad_VOI^PE^. The calibration curves for the three radiomic signatures in predicting p53 status are illustrated in Figure 4b,c, demonstrating good agreement between predicted and actual observations in both cohorts (Mean absolute error = 0.054 and 0.069, Hosmer–Lemeshow test *p* = 0.910 and 0.860, respectively). The DCA plots indicated that the clinical‐radiomics nomogram was the most beneficial prediction model across the entire risk threshold range, highlighting the incremental value of integrating different information sources for clinical application (Figure 4d).

## 4 Discussion

This current study evaluated the ability of CE‐T1WI‐based radiomic signature to predict p53 mutation status noninvasively in IDH‐mt gliomas. The findings affirmed that texture features can depict tumor heterogeneity and suggested the potential of texture analysis at the peritumoral region in identifying p53 mutation status. Moreover, the integrated nomogram combining age and rad‐scores demonstrated superior predictive ability compared to individual radiomic signatures.

Studies have shown that IDH status can predict the prognosis of glioma patients and their response to radiotherapy and chemotherapy [14, 15]. Patients with IDH‐mt LGGs demonstrate improved response to chemotherapy compared to those with IDH‐wt tumors, leading to significantly improved overall survival. The majority of IDH mutations co‐occur with p53 mutations, indicating that IDH mutations are among the earliest events in the pathogenesis of invasive glioma [26, 27]. Molecular subclassification of IDH‐mt gliomas remains challenging in noninvasive diagnosis prior to surgery. Fouke et al. [28] suggested that patients with a suspected LGG should be first scanned using CE‐T1WI, T1WI, T2WI, and FLAIR, which are commonly utilized in clinical practice. Among them, CE‐T1WI is particularly valuable for glioma diagnosis [10]. Previous studies [29, 30] have shown that texture features extracted from CE‐T1WI are particularly informative for glioma grading when compared to features derived from other MRI sequences. Thus, our study focuses on the noninvasive preoperative prediction of p53 status in IDH‐mt gliomas using CE‐T1WI.

In our study, various feature selection methods were used to identify the most discriminative features of p53 status. This approach also tentatively reveals the biological relationship between these features and p53 status. It has been reported that microvascular density is higher in p53‐mt tumors compared to p53‐wt tumors due to the overexpression of EGFR in the latter [14]. Differences in microvascular density can lead to variations in CE‐T1WI signals because of contrast enhancement differences [13], which could explain why median signal intensities are higher in p53‐mt tumors than in p53‐wt tumors. Moreover, p53 mutations promote tumor malignancy and heterogeneity, resulting in consistent differences in expression [31]. Therefore, similar to findings from previous studies [31, 32], our study suggests that uniformity can be used as a predictor of the p53 status in gliomas.

Our analysis of several recent studies has unveiled new associations between radiological characteristics and glioma expression profiles. Furthermore, based on the findings of prior research, three prediction models were devised for tumor regions. In a previous study of glioblastoma, tumors exhibiting high positivity for p53 mutations typically displayed well‐defined lesions with circular enhancement patterns in CE‐T1WI [33]. However, among the three prediction models, the radiomic signature from VOI^T^ exhibited poor predictive performance, with an AUC of 0.770 in both the training and validation cohorts. This predictive efficacy is comparable to that of the model constructed by Li et al. [32] (Table 4), which was based on T2WI sequence images combined with the LASSO algorithm. The predictive efficacy of CE‐T1WI may be similar to that of T2WI in predicting the p53 status of glioma. Another study showed that the AUC of the T2WI‐based p53 prediction model was 0.709 in 10‐mm peritumoral areas [34] (Table 4). Similarly, the performance of the VOI^PE^ signature displayed the most outstanding performance. These results suggest the potential texture value of the tumor margin in identifying p53 mutation status and indicate the inclination of p53‐mt tumors to invade peripherally. In this present study, the predictive value of VOI^T + PE^, which includes the tumor and peritumoral edema, was inferior to that of VOI^PE^ alone. One possible explanation is that within the entire VOI^T + PE^, the intratumoral region influences the strong gray contrast in the peritumoral edema, thus affecting the overall gray statistics. However, the results collectively confirmed that the CE‐T1WI radiomic signatures can serve as a tool for noninvasive prediction of p53 mutation status in IDH‐mt glioma.

**TABLE 4:** Comparison of results with other recent literature.

Study	Models		AUC	Sensitivity	Specificity
Present study	Training cohort	VOI^T^	0.770	0.676	0.786
		VOI^PE^	0.811	0.784	0.643
		VOI^T + PE^	0.846	0.919	0.786
		Nomogram	0.969	0.865	1.000
	Validation cohort	VOI^T^	0.770	0.619	0.667
		VOI^PE^	0.810	0.619	1.000
		VOI^T + PE^	0.778	0.762	0.667
		Nomogram	0.929	0.667	1.000
Li et al. [29]	Training set		0.896	0.803	0.846
	Validation set		0.763	0.622	0.851
Sun et al. [31]	T1 solid		0.673	0.636	0.800
	T2 solid		0.382	0.636	0.400
	T2 5 mm		0.527	0.727	0.200
	T2 10 mm		0.709	1.00	0.400
	T2 15 mm		0.381	0.636	0.200
	T2 20 mm		0.400	0.909	0.200

In addition to radiologic analysis, baseline clinical factors were also considered. Stepwise logistic regression analysis identified age as the only clinical risk factor included in the final model. Stepwise logistic regression analysis identified age as the only clinical predictor (Odds ratio, 0.87; 95% CI: 0.737–1.023). This finding may be influenced by several factors, including the following: First, IDH‐mutant glioma patients are predominantly young and middle‐aged, with an age range of 15 to 47 years [35]; Second, the limited sample size in our study may have increased the risk of selection bias, potentially leading to findings that are not fully representative of the broader patient population. In a previous study, Fu et al. [12] found higher mutant p53 prevalence in high‐grade gliomas compared to low‐grade ones, implying its association with glioma malignancy. Moreover, a study indicated that the prognostic effects of P53 alterations are dependent on patient age [36]. Additionally, age‐related changes in cellular senescence and DNA repair mechanisms can further impact the development and progression of gliomas. Therefore, we cannot dismiss the association between age and p53 status in IDH‐mt gliomas in its entirety. The combination of age and rad‐scores from the three radiomic signatures provided better discrimination compared to a single model in both the training and validation cohorts. The extensive integration of information from diverse sources can enhance the efficacy of clinical decision‐making. Decision curve analysis (DCA) indicated that, when the predicted probability threshold for diagnosing IDH‐mutated glioma with a p53 mutation was established at 60%, the clinical‐radiomics prediction model would result in a net benefit for 80 out of every 100 patients evaluated. Visual nomograms can provide intuitive assessment tools in clinical practice, making prediction results more convenient and readable. To date, no nomogram has been reported for predicting the p53 status of glioma. Therefore, our proposed clinical‐radiomics nomogram may be a valuable tool to assist clinicians in treatment decision‐making, potentially facilitating personalized treatment regimens for glioma patients.

While our results are promising, several limitations should be noted. First, due to the challenge of acquiring a substantial number of available glioma cases at our institution, only 78 cases were included in our study. In our study, we aimed to ensure the highest possible accuracy by including only patients who had undergone genetic sequencing. However, due to the high cost of genetic sequencing, many patients opted for immunohistochemical methods instead. As a result, a significant number of patients were excluded from our analysis. We also excluded cases with low‐contrast enhancement gliomas to ensure the robustness and reliability of our segmentation process. Pertinently, we mitigated this by creating a validation cohort to ensure the validity of our analysis. Second, some cases in our dataset lacked multiple MRI sequence images due to incomplete retrospective data collection, which precluded comprehensive joint analysis and comparison of other MRI sequences. CE‐T1WI is particularly effective in highlighting vascular and enhancing characteristics, making it a preferred sequence for our analysis. To maintain consistency and avoid introducing bias due to missing data, we focused on CE‐T1WI, which was consistently available across our patient population. Third, the limited availability of laboratory tests related to glioma diagnosis meant that only three clinically relevant indicators—age, sex, and pathological stage—were included. Future studies should focus on conducting large‐scale prospective and multi‐center validation cohort collection studies.

## 5 Conclusion

In conclusion, this study's findings suggest that the CE‐T1WI‐based radiomic signature could noninvasively predict the p53 mutation status in IDH‐mutant gliomas of varying grades. The texture variances in peritumor edema may better reflect the underlying tumor heterogeneity associated with p53 status. Furthermore, the combined analysis of a significant clinical factor (age) and radiomic predictors offers a more practical tool for clinical decision‐making in gliomas.

## Author Contributions

**Jiamin Li:** conceptualization (equal), resources (equal). **Zhihong Lan:** conceptualization (equal), resources (equal). **Xiao Zhang:** data curation (equal), software (equal). **Xiaoyun Liang:** data curation (equal), software (equal). **Hanwei Chen:** project administration (equal). **Xiangrong Yu:** project administration (equal).

## Ethics Statement

The Ethics Committee of Zhuhai People's Hospital (approval number: 20220122103) board approval was obtained for this retrospective study, with a waiver of written informed consent from the patients.

## Conflicts of Interest

The authors declare no conflicts of interest.

## Supporting information


**Table S1.** Radiomics features.

## Data Availability

Data available in article Supporting Information.
