# Supplementary material for: Predicting p53 Status in IDH‐Mutant Gliomas Using MRI‐Based Radiomic Model
Source: Cancer Med. 2025 Aug 1;14(15):e71063. doi: 10.1002/cam4.71063 (PMC12314547; doi:10.1002/cam4.71063)
Supplement: Supplementary file 1 — Table S1. Radiomics features. [file CAM4-14-e71063-s001.docx]

**Supplementary note 1: Patient inclusion and exclusion**

Patient inclusion criteria are as follows: 1) patients with available pathological analysis report; 2) patients over 18 years old; 3) patients without surgery, radiotherapy or chemotherapy.

Patients were excluded due to the following conditions: i) patients with Oligodendroglioma, Astrocytoma, and Glioblastoma;ii)patients lack of IDH or p53 gene expression status assessments; iii) patients lack contrast-enhanced T1-weighted (CE-T1WI) and iv) MR images that have motion or other types of artifacts that may affect subsequent segmentation and analysis.

**Supplementary note 2: Customized extraction settings and types of radiomics features**

In this study, we used PyRadiomics v3.0 (on Python 3.6.8) to extract 962 radiomics features from each volume of interest of original and derived MR images. The customized extraction settings were as below:

i) Image type: ‘Original’, ‘LoG’, ‘Wavelet’

For the two kinds of filters, laplacian of Gaussian (LoG) filter emphasizes areas of gray level change and defines fine or coarse textures by the size of sigma, where the values of 2 mm, 4 mm, and 6 mm were explored, respectively. Wavelet filter was applied to focus features on the different decomposition and approximation level of the original contoured volumes, and the bin width of which was set as 10.

ii) Parameter setting: ‘geometryTolerance’, ‘binWidth’, ‘interpolator’, ‘resampledPixelSpacing’

The geometry tolerance was set as 1e-4. Voxels in each volume were resampled to a unified voxel size of 1×1×1 mm^3^ using ‘sitkBSpline’ while the bin width was set as 25.

iii) Feature Class: ‘shape’, ‘firstorder’, ‘glcm’, ‘glrlm’, ‘glszm’, ‘ngtdm’

The feature type and name are listed in Table S1. See <https://pyradiomics.readthedocs.io> for more detailed descriptions and mathematical formulas for these features.

| **Table S1.** Radiomics features | | | |
| --- | --- | --- | --- |
| Feature class | Number | Feature name | |
| Shape | 13 | Flatness | Mesh volume |
|  |  | Least axis length | Minor axis length |
|  |  | Major axis length | Sphericity |
|  |  | Maximum 2D diameter (Column) | Surface area |
|  |  | Maximum 2D diameter (Row) | Surface area to volume ratio |
|  |  | Maximum 2D diameter (Slice) | Voxel volume |
|  |  | Maximum 3D Diameter |  |
| First-order | 18 | 10th percentile | Median |
|  |  | 90th percentile | Minimum |
|  |  | Energy | Range |
|  |  | Entropy | Robust mean absolute deviation |
|  |  | Interquartile range | Root mean squared |
|  |  | Kurtosis | Skewness |
|  |  | Maximum | Total Energy |
|  |  | Mean absolute deviation | Uniformity |
|  |  | Mean | Variance |
| GLCM | 24 | Auto correlation | Inverse difference normalized |
|  |  | Cluster prominence | Informational measure of correlation1 |
|  |  | Cluster shade | Informational measure of correlation2 |
|  |  | Cluster tendency | Inverse variance |
|  |  | Contrast | Joint average |
|  |  | Correlation | Joint energy |
|  |  | Difference average | Joint entropy |
|  |  | Difference entropy | Maximal correlation coefficient |
|  |  | Difference variance | Maximum probability |
|  |  | Inverse difference | Sum average |
|  |  | Inverse difference moment | Sum entropy |
|  |  | Inverse difference moment Normalized | Sum squares |
| GLRLM | 16 | Gray level non-uniformity | Run entropy |
|  |  | Gray level non-uniformity Normalized | Run length non-uniformity |
|  |  | Gray level variance | Run length non-uniformity Normalized |
|  |  | High gray level run emphasis | Run percentage |
|  |  | Long run emphasis | Run variance |
|  |  | Long run high gray level Emphasis | Short run emphasis |
|  |  | Long run low gray level Emphasis | Short run high gray level Emphasis |
|  |  | Low gray level run emphasis | Short run low gray level Emphasis |
| GLSZM | 16 | Gray level non-uniformity | Size-zone non-uniformity |
|  |  | Gray level non-uniformity Normalized | Size-zone non-uniformity Normalized |
|  |  | Gray level variance | Small area emphasis |
|  |  | High gray level zone emphasis | Small area high gray level Emphasis |
|  |  | Large area emphasis | Small area low gray level Emphasis |
|  |  | Large area high gray level Emphasis | Zone entropy |
|  |  | Large area low gray level Emphasis | Zone percentage |
|  |  | Low gray level zone emphasis | Zone variance |
| NGTDM | 5 | Busyness | Contrast |
|  |  | Coarseness | Strength |
|  |  | Complexity |  |
| Abbreviations: *GLCM,* gray level co-occurrence matrix; *GLRLM,* gray level run length matrix; *GLSZM,* gray level size zone matrix; *NGTDM,* neighboring gray tone difference matrix | | | |
